# Assuring Bangladesh’s future: non-communicable disease risk factors among the adolescents and the existing policy responses

**DOI:** 10.1186/s41043-022-00294-x

**Published:** 2022-05-16

**Authors:** Tuhin Biswas, Peter Azzopardi, Syeda Novera Anwar, Tim David de Vries, Luis Manuel Encarnacion-Cruz, Md. Mehedi Hasan, M. Mamun Huda, Sonia Pervin, Rajat Das Gupta, Dipak Kumar Mitra, Lal B. Rawal, Abdullah Al Mamun

**Affiliations:** 1 Institute for Social Science Research, The University of Queensland, QLD, Australia; 2 ARC Centre of Excellence for Children and Families Over the Life Course, The University of Queensland, QLD, Australia; 3 Burnet Institute, Melbourne, VIC Australia; 4 Institute of Health Economics, University of Dhaka, Dhaka, Bangladesh; 5 NCD Alliance, Geneva, Switzerland; 6 Utrecht University, Utrecht, Netherlands; 7 Maternal and Child Health Division (MCHD), icddr,b, Dhaka, Bangladesh; 8 Department of Epidemiology and Biostatistics, Arnold School of Public Health, University of South Carolina, Columbia, SC USA; 9 Department of Public Health, North South University, Dhaka, Bangladesh; 10 School of Health, Medical and Applied Sciences, CQ University, Sydney, Australia

**Keywords:** Non-communicable disease, Adolescents, Health policy, Bangladesh

## Abstract

**Background:**

The aim of this study is to assess the current status of non-communicable disease (NCD) risk factors amongst adolescents in Bangladesh. We also critically reviewed the existing policy responses to NCD risk among adolescents in Bangladesh.

**Methods:**

This study used a mixed method approach. To quantify the NCD risk burden, we used data from the Global School-based Student Health Survey conducted in Bangladesh. To understand policy response, we reviewed NCD-related policy documents introduced by the Government of Bangladesh between 1971 and 2018 using the WHO recommended NCD Action Plan 2013–2020as study framework. Information from the policy documents was extracted using a matrix, mapping each document against the six objectives of the WHO 2013–2020 Action Plan.

**Results:**

Almost all adolescents in Bangladesh had at least one NCD risk factor, and there was a high prevalence of concurrent multiple NCD risk factors; 14% had one NCD risk factor while 22% had two, 29% had three, 34% had four or more NCD risk factors. Out of 38 policy documents, eight (21.1%) were related to research and/or surveys, eight (21.1%) were on established policies, and eleven (29%) were on legislation acts. Three policy documents (7.9%) were related to NCD guidelines and eight (21.1%) were strategic planning which were introduced by the government and non-government agencies/institutes in Bangladesh.

**Conclusions:**

The findings emphasize the needs for strengthening NCD risk factors surveillance and introducing appropriate intervention strategies targeted to adolescents. Despite the Government of Bangladesh introducing several NCD-related policies and programs, the government also needs more focus on clear planning, implementation and monitoring and evaluation approaches to preventing NCD risk factors among the adolescents in Bangladesh.

**Supplementary Information:**

The online version contains supplementary material available at 10.1186/s41043-022-00294-x.

## Introduction

Adolescents represent almost a quarter of the world’s population [1], and adolescence is now recognised as  a pivotal developmental stage where investments can result in healthier young people, healthier adults and a healthier next generation [2, 3]. Nearly 35% of the global burden of disease has its origins in adolescence, and more than 3000 adolescents die every day, mostly due to non-communicable diseases (NCDs), intentional and unintentional injuries and other preventable causes [4, 5].

NCD-related deaths are increasing, especially in low- and middle-income countries (LMICs) [6] and over half of these deaths are associated with risk that emerge during the adolescence. These include, but not limited to tobacco and alcohol use, poor diet, and insufficient physical activity [6]. According to the Lancet Commission on Adolescent Health (2016), tobacco use, alcohol consumption, overweight, obesity, and mental health problems were identified as the major health risks for adolescents around the world [7, 9]. Early tobacco use is a major risk factor for NCDs throughout the life. In addition, smoking at a young age also increases the risk of many diseases among adolescents such as respiratory illness, asthma, and reduced pulmonary function [6]. Alcohol consumption is linked to development of different types of cancers, hypertension, hemorrhagic stroke and other NCDs [8, 10–12]. Numerous studies have reported that overweight and obesity are increasing markedly across adolescence and young adulthood [13]. To combat the burden of NCDs worldwide, the Sustainable Development Goals (SDGs) include a specific target for reducing premature death from NCDs by one-third, through prevention and treatment and promotion of mental health and well-being by 2030. However, the importance of NCD prevention among adolescents in global NCD declarations and action plansis yet to be fully realised.

Like many LMICs, Bangladesh is undergoing rapid urbanization, which has triggered changes in population dynamics and their disease patterns. The country is at an advanced phase of the third stage of the epidemiological transition, which means that deaths from NCDs are expected to increase rapidly in the coming years [14, 15]. Adolescents account  for  one third of the country's total population. A recent study in Bangladesh reported that 18% of the adolescents had three or more risk factors, with males reporting higher prevalence than females [4, 5]. Another study by Urmy et al. reported that younger age, non-slum urban and slum residence, higher paternal education, and depression were associated with the coexistence of multiple risk factors among the adolescent [6]. However, no study has been conducted in in Bangladesh which provides comprehensive details on NCD risk factors among adolescents and its policy response.

Bangladesh is categorized as a ‘multi-burden country’—i.e., high rate of Communicable/maternal/nutritional, high rate of injury,  and a high rate of NCDs for adolescents [7, 9]. The government of Bangladesh has duly recognized this complex profile of need and has formulated a number of policy documents and strategies to address the NCD burden and associated risk factors. However, the evidence of the effective implementation of these policies is limited. A comprehensive analysis of the existing policies is warranted to ascertain the adequacy of national straggles to tackle the adolescent NCDs,

In order to determine the current status of NCD risk factors among the adolescents in Bangladesh, we analyzed the Global School-based Health Survey (GSHS). We also examined the existing national policies and strategies through the lens of the World Health Organization’s 2013–2020 Action Plan for the Global Strategy for the Prevention and Control of NCDs. This policy analysis determined the extent to which the objectives of the WHO Action Plan have been met at national policy level.

## Methods

### Data source

#### Quantitative data

The quantitative data presented in this study come from the Global School-based Health Survey (GSHS), which was conducted for the first time in Bangladesh in 2014 [16]. The 2014 Bangladesh GSHS was administered among adolescents aged 12–17 years to capture information on a wide range of health indicators. The 2014 Bangladesh GSHS employed a two-stage cluster sampling technique. At the first stage, the schools were selected randomly from a list of schools. Classes that provide a representative sample of the general population aged 12–17 years were selected within the selected schools at the second stage of sampling.

#### Policy documents

We also reviewed different policy documents on adolescent health issue. We considered if these  policy documents were  aligned directly or indirectly with the prevention and control of NCD risk factors among the adolescents in Bangladesh. Different search engines such as PubMed and Google Scholar were used to identify relevant documents. The key terms used in the website searches were ‘adolescent health,’ ‘health education,’ ‘mental health,’ ‘nutrition for adolescents,’ ‘violence against adolescents,’ combined with ‘policy,’ ‘action plan,’ ‘strategy,’ ‘guideline and “Bangladesh”.’ The search strategy employed with PubMed is as follows:

((((("adolescent health"[MeSH Terms] OR ("adolescent"[All Fields] AND "health"[All Fields]) OR "adolescent health"[All Fields]) OR ("mental health"[MeSH Terms] OR ("mental"[All Fields] AND "health"[All Fields]) OR "mental health"[All Fields])) OR (("nutritional status"[MeSH Terms] OR ("nutritional"[All Fields] AND "status"[All Fields]) OR "nutritional status"[All Fields] OR "nutrition"[All Fields] OR "nutritional sciences"[MeSH Terms] OR ("nutritional"[All Fields] AND "sciences"[All Fields]) OR "nutritional sciences"[All Fields]) AND ("adolescent"[MeSH Terms] OR "adolescent"[All Fields] OR "adolescents"[All Fields]))) OR (("violence"[MeSH Terms] OR "violence"[All Fields]) AND against[All Fields] AND ("adolescent"[MeSH Terms] OR "adolescent"[All Fields] OR "adolescents"[All Fields]))) AND (((("guideline"[Publication Type] OR "guidelines as topic"[MeSH Terms] OR "guideline"[All Fields]) OR strategy[All Fields]) OR (("United Evangelical Action"[Journal] OR "Action Natl"[Journal] OR "action"[All Fields]) AND plan[All Fields])) AND ("policy"[MeSH Terms] OR "policy"[All Fields]))) AND ("Bangladesh"[MeSH Terms] OR "Bangladesh"[All Fields]).

We also searched the gray literature in the Demography and Health Survey (DHS) database, WHO regional databases and Global Burden of Diseases (GBD) database and Bangladesh government and ministries home pages. Response rate was 91%.

### Measurement

#### Quantitative data

GSHS capture   information on a wide range of health indicators, including insufficient physical activity, alcohol consumption, any form of tobacco, sedentary behavior, insufficient fruits and vegetables consumption, overweight/obesity and psychological distress. Details of each measurement are described in Additional file 1.

*For policy analysis,* a pre-structured case methodology was adopted, using an existing conceptual NCD framework to define the structure for data collection and analysis [17]. The framework used the six objectives of the WHO Action Plan as listed in Additional file 2. The scope of the analysis is limited to the policies relevant to adolescent NCDs.

### Data analysis

The data analysis was performed on a total of 2,989 students. Data were analyzed using the IBM SPSS (version 20) software. All analyses were adjusted for sample weight. Policy documents that are available in public domain and were published between 1971 and 2018 were identified through online searches in PubMed and Google Scholar. Extensive review of existing policies relevant to prevention and control of NCDs in Bangladesh was done in an earlier publication by using data display matrix. Furthermore, we will now focus on the policy documents that may have direct or indirect impact on the health systems preparedness to tackle NCDs among Bangladeshi adolescents.

### Ethical consideration

In each of the participating countries, the GSHS received ethics approval from the Ministry of Education or a relevant Institutional Ethics Review Committee, or both. Only adolescents and their parents who provided written or verbal consent participated. As the current study used retrospective publicly available data, we did not need ethics approval from any Institutional Ethics Review Committee.

### Patient and public involvement

Patients were not involved in the study. The survey covered school-based young adolescents aged between 12 and 17 years old.

## Results

### Findings from the secondary analyses of GSHS data

#### Prevalence of NCD risk factors among the adolescents

**Fig. 1:**
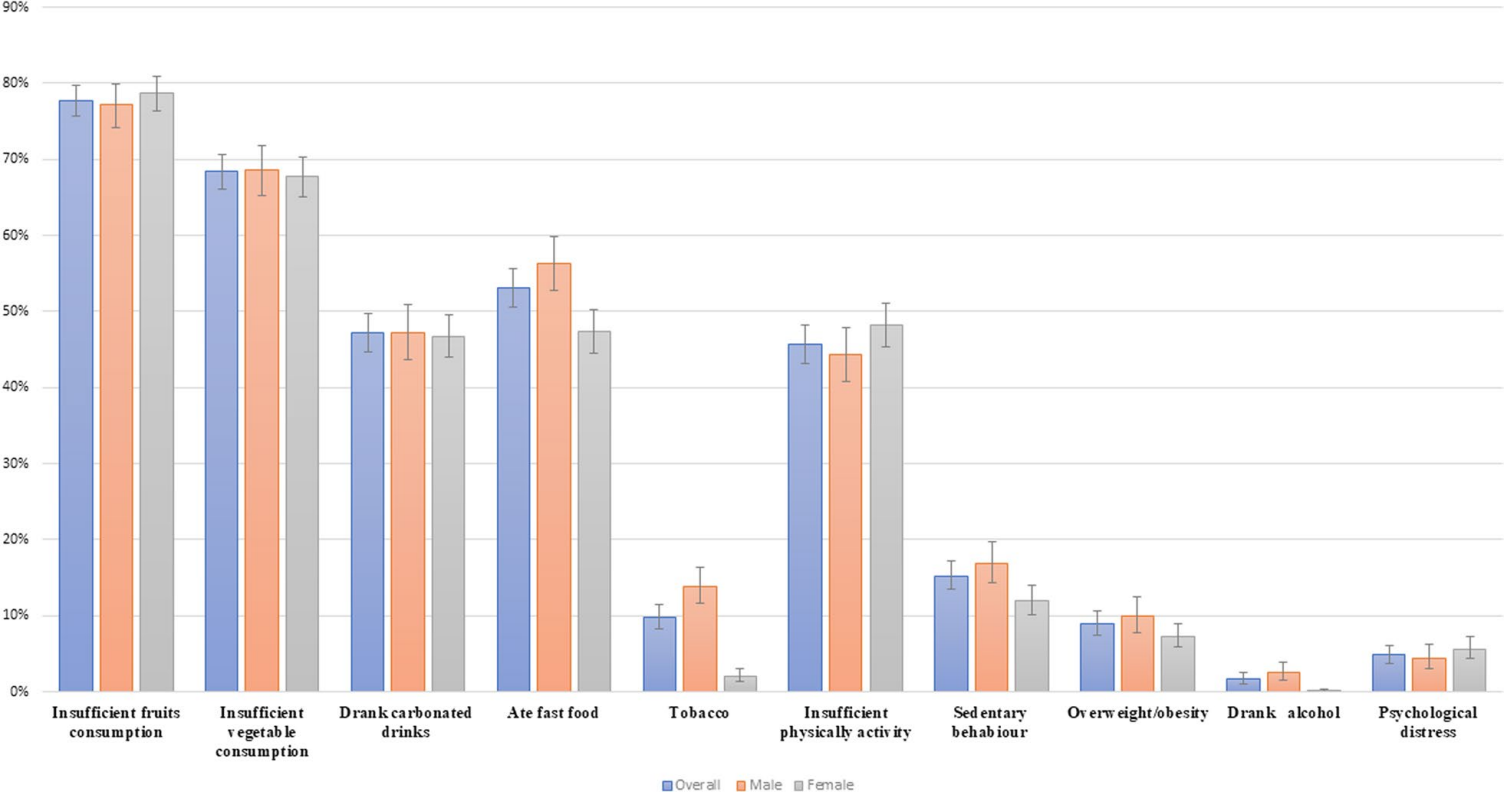
NCD risk factors among the adolescents in Bangladesh

Of the 2,972 adolescents aged 12–17 years, 1191 (40.07%) were males and 1781 (59.93%) were females. Figure 1 shows the prevalence of different NCD risk factors among the adolescents by gender. Insufficient vegetables consumption (boys: 69%, 95% CI 65–%-71%; girls: 68%, 95% CI 65–70%; *p* = 0.46)*,* insufficient fruits consumption (boys: 77%, 95% CI 74–79% vs girls: 79%, 95% CI 76–80%; *p* = < 0.005), ate fast food (boys: 56%, 95% CI: 52–59% vs girls: 47%, 95% CI 44–50%; *p* =  < 0.005), insufficient physically activity (boys: 44%, 95% CI 40–47% vs girls: 48%, 95% CI 45–51%; *p* = < 0.005) and psychological distress (boys: 4%, 95% CI 2–5% vs girls: 6%, 95% CI 4–7%; *p* =  < 0.005) were found to be similar for adolescent boys and girls. Prevalence of alcohol consumption, used any form of tobacco and overweight/obesity were higher among the boys compared to the girls. Use of any form of tobacco was more than seven times higher among the boys compared to the girls.

**Fig. 2:**
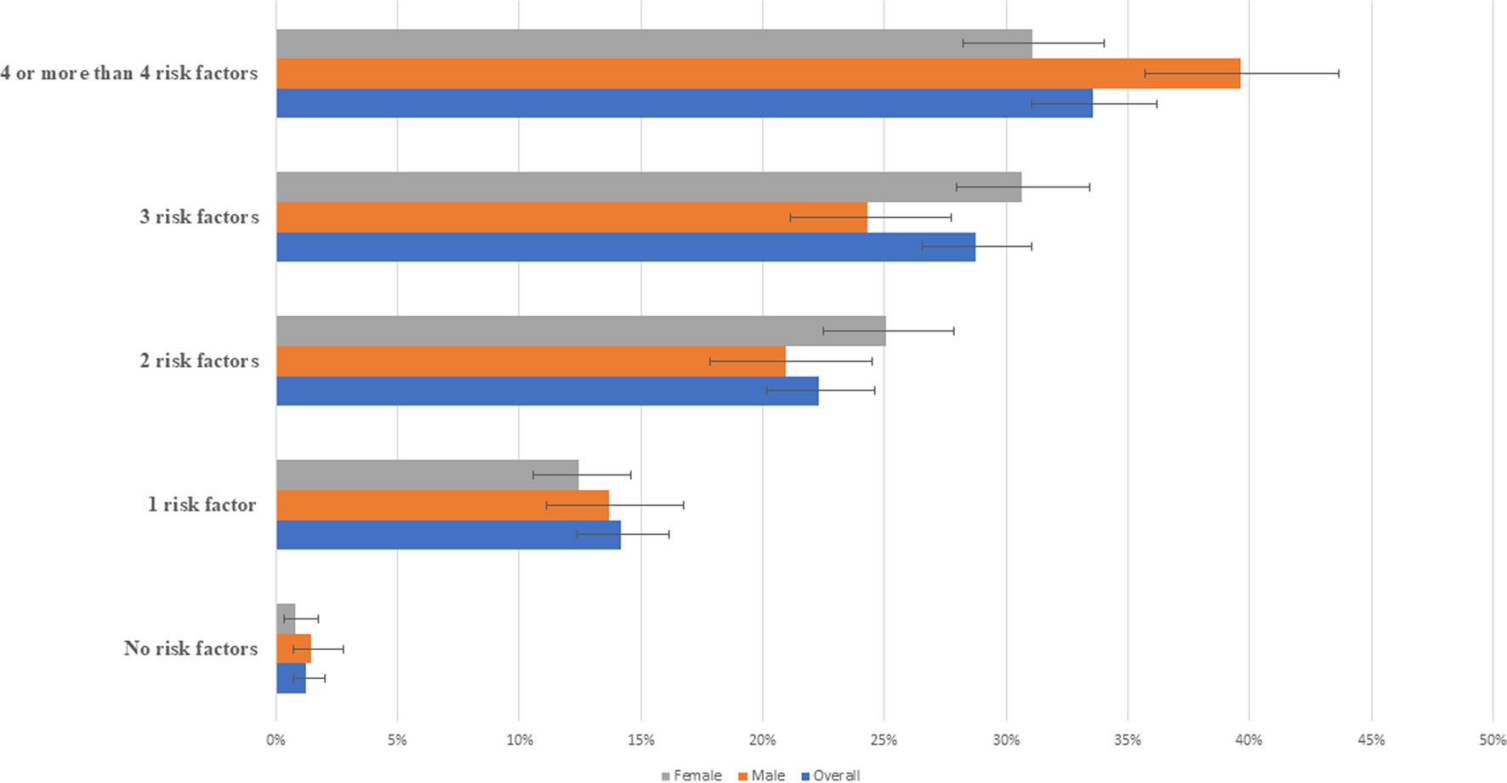
Clustering NCD risk factors among the adolescents in Bangladesh

We examined the clustering (presence of multiple risk factors in an individual) of NCD risk factors in the sample and have analyzed by gender. According to Fig. 2, only 1% adolescent did not have any NCD risk factor. Overall 14% had at least one risk factor, 22% had two risk factors, 29% had three risk factors and 34% had four or more risk factors.

### Findings from the review of policy documents related to adolescents NCD risk

#### Policy response

**Fig. 3:**
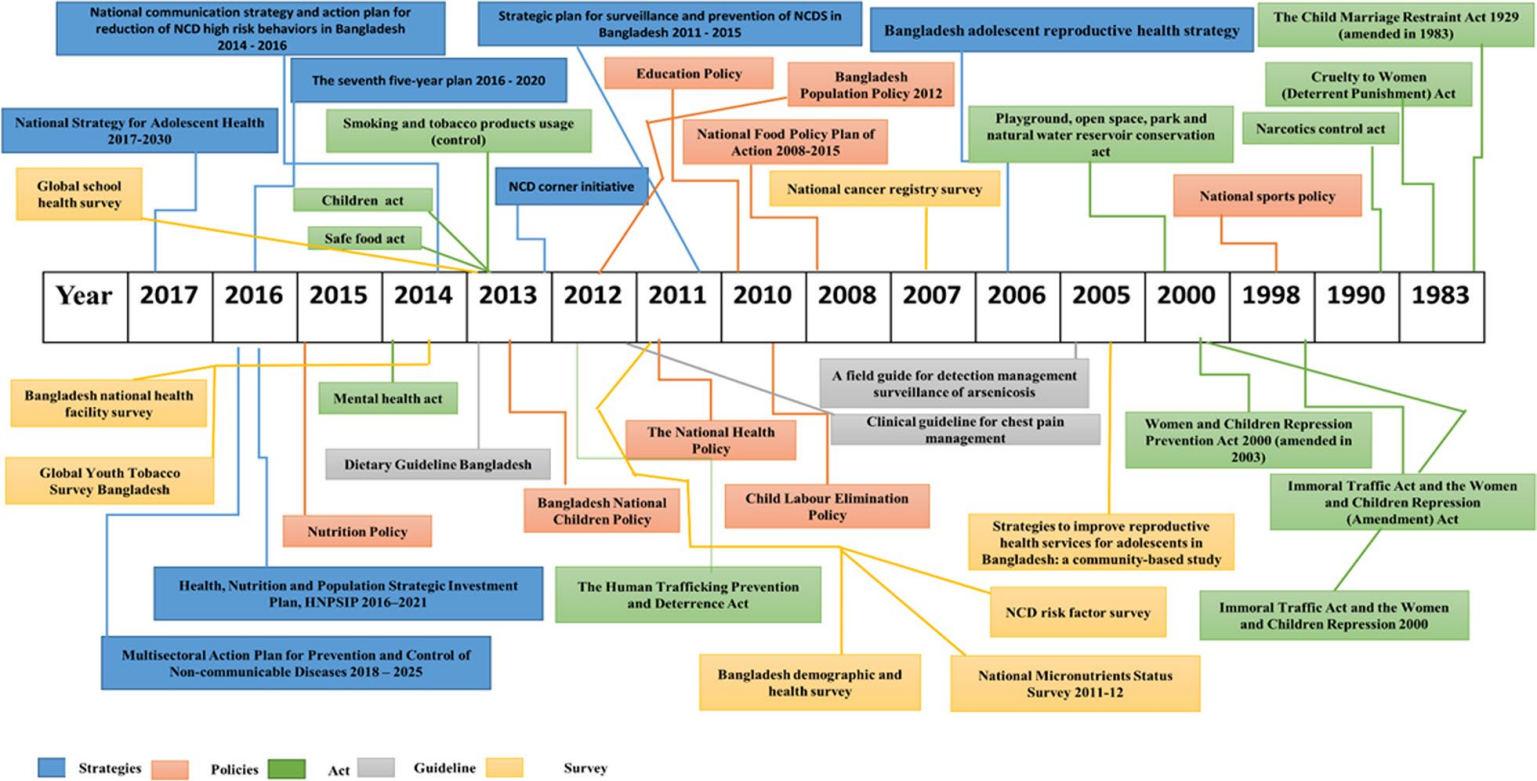
Timeline of major policy formulation and program initiatives relevant to adolescent NCDs in Bangladesh

A total of 38 documents were identified [16, 18–54]. Eight (21.1%) were research and/or surveys, eight were on established policies (21.1%), while eleven (29%) were on acts. Three (7.9%) were related to guidelines and eight (21.1%) were strategic planning documents from government and non-government agencies/institutes **(**Fig. 3). Table 1 presents the summary of the abovementioned policy documents against the objectives of the WHO Action Plan.

**Table 1:** Summary of policy documents for each objective of the Action Plan

List of policy documents and published year	Objective 1	Objective 2	Objective 3	Objective 4	Objective 5	Objective 6
*Strategies*						
Bangladesh adolescent reproductive health strategy (2006) [18]	√	√	√	√	√	√
Strategic plan for surveillance and prevention of NCDs in Bangladesh 2011–2015 (2011) [19]	√	√	√	√	√	√
NCD corner initiative (2013) [20]	×	√	√	√	√	√
National communication strategy and action plan for reduction of NCD high risk behaviours in Bangladesh 2014–2016 (2014) [21]	×	√	√	√	√	√
The seventh 5-year plan 2016–2020 (2016) [22]	×	√	√	√	√	√
Health, Nutrition and Population Strategic Investment Plan, HNPSIP 2016–2021 (2016) [23]	×	√	√	√	√	√
The multi-sectoral action plan for the NCD control and prevention 2018–2025 (2018) [24]	×	√	√	√	√	√
National Strategy for Adolescent Health 2017–2030 (2017) [25]	√	√	√	√	√	√
*Policies*						
National sports policy (1998) [26]	√	√	√	√	√	×
National Food Policy Plan of Action 2008–2015 (2008) [27]	√	√	√	√	√	×
Education Policy (2010) [28]	×	√	√	√	√	×
Child Labour Elimination Policy (2010) [29]	×	√	√	√	√	×
The National Health Policy (2011) [30]	√	√	√	√	√	×
Bangladesh Population Policy (2012) [31]	×	√	√	√	√	×
Bangladesh National Children Policy (2011) [32]	√	√	√	√	√	×
Nutrition Policy (2015) [33]	√	√	√	√	√	×
*Legislation Acts*						
The Child Marriage Restraint Act 1929 (amended in 1983) (1929) [34]	×	√	√	×	×	×
Cruelty to Women (Deterrent Punishment) Act (1983) [35]	×	√	√	×	×	×
Narcotics control act (1990) [36]	×	√	√	×	×	×
Playground, open space, park and natural water reservoir conservation act (2000) [37]	×	√	√	×	×	×
Prevention of Oppression against Women and Children Act 2000 (amended in 2003) [38]	×	√	√	×	×	×
Immoral Traffic Act (2010) [39]	×	√	√	×	×	×
The Human Trafficking Prevention and Deterrence Act (2012) [40]	×	√	√	×	×	×
Safe food act (2013) [41]	×	√	√	×	×	×
Smoking and tobacco products usage (control) (amendment) Act (2013) [42]	×	√	√	×	×	×
Children Act (2013) [43]	×	√	√	×	×	×
Mental health act (2014) [44]	×	√	√	×	×	×
*Guideline*						
A field guide for detection management surveillance of arsenicosis (2005) [45]	×	√	×	√	×	×
Acute chest pain management protocol (2012) [46]	×	√	×	√	×	×
Dietary Guideline Bangladesh (2013) [47]	×	√	×	√	×	×
*Surveys and researches*						
Strategies to improve reproductive health services for adolescents in Bangladesh: a community-based study (2005) [48]	×	×	×	×	√	√
National cancer registry survey (2007) [49]	×	×	×	×	√	√
Global Youth Tobacco Survey Bangladesh (2013) [50]	×	×	×	×	√	√
Bangladesh demographic and health survey (2011) [51]	×	×	×	×	√	√
NCD risk factor survey (2011) [52]	×	×	×	√	√	√
National Micronutrients Status Survey 2011–12 (2011) [53]	×	×	×	×	√	√
Bangladesh national health facility survey (2014) [54]	×	×	×	×	√	√
Global school health survey (2014) [16]	×	×	×	×	√	√
Total	8	30	27	20	24	16

##### Objective 1: prioritize NCDs prevention and control

This objective was addressed by eight documents, among which three were strategy papers [18, 19, 25] and five were policy papers (Table 1) [26, 27, 30, 32, 33]. Government of Bangladesh already implemented a 10-year long adolescent reproductive health strategy from (ARHS) 2006–2016 [18]. After completing the period of ARHS, government has recently formulated and started to implement the National Strategy for Adolescent Health 2017–2030 with the alignment of sustainable development goals. This is supposed to be a comprehensive strategy covering all dimensions to ensure the proper wellbeing of adolescents. Adolescent sexual and reproductive health, violence against adolescents, adolescent nutrition and mental health of adolescents are identified as four strategic thematic areas for this strategy, including two cross-cutting issues of social and behavioral change communication and health systems strengthening. But in this strategy, most of the NCD risk factors among the adolescent overlooked.

##### Objective 2: strengthen national capacity and multi-sectoral action

This objective was addressed by thirty documents. Of whom, eight were strategy papers [18–25], eleven were policy acts [34–44], three were guidelines [45–47] and eight were policy papers (Table 1) [26–33]. Both national and international agencies extend their financial and technical support to various health promotion programs and researches targeted toward particular age groups, e.g., the adolescents.

##### Objective 3: reduce modifiable risk factors for NCDs

This objective was addressed by twenty seven documents, among which eight were strategy papers [18–25], eight were policy papers [26–33] and eleven were acts [34–44] (Table 1). The “Smoking and Tobacco Products Usage (Control) Act” of 2005 had addressed many issues regarding this objective. The act was amended in 2013 and had strictly restricted the harmful use of tobacco products by all age groups, especially the adolescents as the purchase and sale of tobacco products to the underage (below the age of 18 years) was banned [39].

##### Objective 4: strengthen health systems

This objective was addressed by twenty documents, among which eight were strategy papers [15–22], eight were policy papers [23–30], one survey [42–44] and three were guideline [42–44] documents (Table 1). The National NCD Risk Factor Survey 2011 had guided several initiatives to tackle NCDs. Non-Communicable Diseases Centre (NCDC) has been established within the Ministry of Health and Family Welfare. NCD corners in 300 Upazila Health Complexes have facilitated the access to health care to the grass root level. However, in the context of health systems structure in Bangladesh, still there is no specific counselling corner for adolescent, especially adolescent’s mental health well-being [20].

##### Objective 5: national capacity for high-quality research

This objective was addressed by sixteen documents, among which eight were strategy papers [18–25], eight were policy papers [26–33] and eight were surveys or research [16, 48–54] (Table 1). The Directorate General of Health Services (DGHS) [52] invests in NCD-related researches. In 2010, the DGHS has conducted the NCD Risk Factor Survey. Later in 2013, the National Center for Control of Rheumatic Fever and Heart Disease had conducted the GATS [50], and the Global School-based Student Health Survey 2014 [16]. NIPORT conducts Bangladesh Demographic and Health Survey at every 4 years interval [51]. International agencies such as WHO and Japan International Cooperation Agency (JICA) also facilitate several surveys with their financial and technical support.

##### Objective 6: monitor the trends and evaluate progress

This objective was addressed by sixteen documents, among which eight were strategy papers [18–25] and eight were surveys or research (Table 1) [16, 48–54]. This objective has been addressed by NCD Risk Factor Survey, GATS, Global School Health survey, and the mental health survey [16, 48–54]. The Global School Health survey had important findings on the nutritional and mental health status of school-going students in Bangladesh [16]. BDHS 2011 had incorporated a few components of NCD. But BDHS 2011 completely ignored adolescents NCDs [51]. Bangladesh health facility survey also described current situation of the respective areas [54].

## Discussion

This study provides the contemporary evidence on NCD risk factors among the adolescent in Bangladesh, and we have presented the policy provisions to combating the NCD risk factors among the adolescents in Bangladesh. The study reported high prevalence of NCD risk factors among the adolescent. More than thirty percent of adolescents had four or more NCD risk factors. Moreover, analysis of the policy documents suggests that, over the years, the government of Bangladesh has undertaken many endeavors to tackle NCDs. Some of the initiatives  demonstrate innovative approach and effectiveness, such as the NCD corners initiative at Upazila Health Complexes. The ban on tobacco product advertisements and plain packaging of tobacco products were also considered to be bold steps toward prevention of a major NCD risk factor. The National Safe Food Act of 2013 promoted food safety for all.

In our study, we found that insufficient fruits, insufficient vegetables and fats food consumption are the three common risk factors among the adolescent. This is similar to an analysis in Vietnam that reported low fruit/vegetable intake and unhealthy diet were common among school-going adolescents aged 13–17 years [7, 9]. Similar to some other Asian countries, we found that smoking and alcohol prevalence is comparatively low in Bangladesh [8, 10]. Although in our study we did not explore  any school health promotion programs for improving adolescent health wellbeing, a recent study in Vietnam reported high quality of health promotion programs associated with lower the odds of lifestyle risk behaviors [7, 9].

According to our study, nearly fifty percent of adolescents are physically inactive. With a vision of NCD prevention from early age, WHO has outlined guidelines for games and physical activities within  educational institutes [8]. Bangladesh has incorporated this vision in the National Children’s Policy, educational policy and the National Sports Policy. Unfortunately, these policies do not reflect on a large number of educational institutions, especially in urban areas, where there is not enough open space for physical activities or sports.

Appropriate nutrition is important for adolescence to secure current and future generation health [32]. In our study, we found that around 10% adolescents were overweight/obese, and 78% and 68% adolescent consumed insufficient fruits and vegetables, respectively. More than fifty percent adolescent ate fast food. It demonstrated that prevalence of unhealthy food habit is increasing, with the trend continuing into early adulthood [33]. This is an impending public health problem that needs further action as adolescent obesity strongly predicts adult obesity and associated morbidity. Addressing this issue at a population level is a critical starting point to avert potential long-term impacts of adolescent obesity [34].

Bangladesh has an adolescent and youth population of approximately 52 million, amounting to 1/3rd of the country's total population. [41]. According our study, NCD risk factors among the adolescent comparatively high. At the same time, the strict enforcement of these policies is still very challenging in country context of Bangladesh. Like many other countries [55] it is challenging to design and implement appropriate actions for the wellbeing of adolescent health. Greater emphasis must also be placed on further enhancing the data collection system with a view to strengthening the Health Management Information System so as to better serve the adolescents.

A recent study in Bangladesh entitled “A scorecard for tracking actions to reduce the burden of non-communicable diseases” reported that among the four domains of governance, risk factor surveillance, research, and health system response, the country’s performance score was low in three domains, except for the governance domain (moderate performance) [56]. In addition to that, the country lacks any integrated community public health program focused on monitoring NCDs amongst adolescents on a regular basis. Bangladesh also lacks any national surveillance program focused on NCDs. Only a few tertiary hospitals maintain such NCD surveillance systems [57] but most of them targeting adult population. Another study identified a total of 11 NCD programs in Bangladesh focusing on tobacco-related illnesses, diabetes or cardiovascular diseases [58]. Unfortunately, to date, there are no such programs being developed to target the NCDs prevention for adolescents in Bangladesh.

### Strength and limitations

The strengths of this study include nationally representative sample taken through standardized questionnaire. To the best of our knowledge, this is a first large population-based study ever been conducted to examine the NCD risk factors among adolescents in Bangladesh. The present study has some limitations. Firstly, data were collected from adolescents enrolled in schools. Secondly, as the questionnaire was self-reported, it is possible that some respondents might have misreported the questions asked. Thirdly, the scope of this policy analysis was limited to publicly available documents in relation to the objectives of the WHO Action Plan.

## Conclusion

Adolescents in Bangladesh report a high prevalence of concurrent multiple risk factors for NCDs. The government of Bangladesh has demonstrated political commitment toward adhering the global action plans to reduce the burden of NCDs in all age groups. However, more planning and coordination of existing programs are warranted with a focus on adolescents. Given the growing burden of NCD risk factors in this age group, it is likely to result in excessive NCDs burden in near future, if no appropriate and immediate actions are taken. Priority should be given on the prevention of modifiable risk factors from an early age. Programs and interventions targeted at educational institutions could provide an integrated platform for NCDs prevention and control. Further research and monitoring of the trends of NCDs among adolescents would guide us toward appropriate strategies to address the NCD burden.

## Supplementary Information

**Additional file 1.** Measurement of NCD risk factors (insufficient physical activity, alcohol consumption, any form of tobacco, sedentary behavior, insufficient fruits and vegetables consumption, overweight/obesity and psychological distress).

**Additional file 2.** Six objectives of the WHO Action Plan.

## Data Availability

We thank the US Centers for Disease Control and WHO for making Global School-based Student Health Survey (GSHS) data publicly available for analysis.

## Abbreviations

NCD: Non-communicable disease
LMICs: Low and middle-income countries
SDGs: Sustainable development goals
GSHS: Global School-based Health Survey
WHO: World Health Organization
CDC: Centre for Disease Control and Prevention
UNAIDS: United Nations Programme on HIV and AIDS
