# Supplementary material for: Assuring Bangladesh’s future: non-communicable disease risk factors among the adolescents and the existing policy responses
Source: J Health Popul Nutr. 2022 May 16;41:22. doi: 10.1186/s41043-022-00294-x (PMC9109415; doi:10.1186/s41043-022-00294-x)
Supplement: Supplementary file 1 — Additional file 1. Measurement of NCD risk factors (insufficient physical activity, alcohol consumption, any form of tobacco, sedentary behavior, insufficient fruits and vegetables consumption, overweight/obesity and psychological distress). [file 41043_2022_294_MOESM1_ESM.docx]

**Supplementary table-1: Measurement of NCD risk factors (insufficient physical activity, alcohol consumption, any form of tobacco, sedentary behavior, insufficient fruits and vegetables consumption, overweight/obesity and psychological distress).**

| **Insufficient physical activity** | Physical activity was calculated according to the number of times physical activity was performed for at least 60-minute sessions in the past seven days, considering any type of physical activity that increased the heart rate and breathing of adolescents. Adolescents who practiced physical activities less than five times per week were considered to have low physically activity**.** |
| --- | --- |
| **Sedentary behavior** | Sedentary behavior was assessed by the total screen time (sum of daily television, computer and video game time) on weekdays and during the weekend. Individuals who spend three or more hours per day in front of the screen were considered to have sedentary behavior and this cut-off point for total screen time has been previously used in several studies. |
| **Any form of tobacco** | To assess any form of tobacco, GSHS considered those who currently smoking and currently used any tobacco product such as chewing tobacco (on at least one day during the past 30 days before the survey). |
| **Insufficient fruits and vegetable intake** | Information was recorded on how many days the respondents have consumed fruits and vegetables in a typical week, and the number of servings of fruits and vegetables consumed on average per day. GSHS used the WHO food frequency questionnaire to assess this indicator. As recommended by WHO [18], the consumption of less than two servings of fruits and three serving’s vegetables per day was classified as insufficient fruit and vegetable intake. |
| **Carbonated soft drinks** | During the past 30 days how many times per day did you usually drink carbonated soft drinks, such as Pepsi, Coca cola, 7-up, Sprite, Fanta and Dew?” (Recoded: I did not drink and <1 times/day as ‘no’ and 1 to >5 times/ day as ‘yes’). |
| **Fast food** | During the past 7 days on how many days did you eat food from a fast food restaurant such as McDonalds, KFC, Pizza Hut, Subway, AFC?” (Recoded: 0 days as ‘no’ and 1 to 7 days as ‘yes’). |
| **Overweight/obesity** | International age- and sex-specific child body mass index (BMI), calculated as weight/height2 (kg/m2) cut-points were used to define overweight and obesity. School going children are categorized as overweight if their BMI Z-score was more than one standard deviation (> +1 SD) from the median BMI for age and sex. |
| **Alcohol consumption** | During the past 30 days, those who reported consuming alcohol on one or more days were considered to have this risky behavior because excessive alcohol consumption even on single occasion can be considered as deleterious to health. |
| **Psychological distress** | Psychological distress was identified by asking the question: “During the past 12 months, did you ever seriously consider attempting suicide?” |
