# Supplementary material for: Assuring Bangladesh’s future: non-communicable disease risk factors among the adolescents and the existing policy responses
Source: J Health Popul Nutr. 2022 May 16;41:22. doi: 10.1186/s41043-022-00294-x (PMC9109415; doi:10.1186/s41043-022-00294-x)
Supplement: Supplementary file 2 — Additional file 2. Six objectives of the WHO Action Plan. [file 41043_2022_294_MOESM2_ESM.docx]

**Not found full text= 2**

**Not relevant to adolescent health= 28**

**Duplicate= 20**

**38 studies included for policy analysis**

**Article included further evaluation= 40**

**Grey literature/Government documents**

**Total= 88**

**Article remaining after duplication= 68**

**PubMed, Goggle and Goggle scholar**

**Supplementary figure-1: Flow diagram of study selection**

**Supplementary table-2: Six objectives of the WHO Action Plan**

| **Objective 1** | To raise the priority accorded to the prevention and control of non-communicable diseases in global, regional and national agendas and that of internationally agreed development goals, through strengthened international cooperation and advocacy. |
| --- | --- |
| **Objective 2** | To strengthen national capacity, leadership, governance, multi-sectoral action and partnerships to accelerate country response for the prevention and control of non-communicable diseases |
| **Objective 3** | To reduce modifiable risk factors for non-communicable diseases and underlying social determinants through creation of health-promoting environment |
| **Objective 4** | To strengthen and orient health systems to address the prevention and control of non-communicable diseases and the underlying social determinants through people-centered primary health care and universal health coverage |
| **Objective 5** | To promote and support national capacity for high-quality research and development for the prevention and control of non-communicable diseases |
| **Objective 6** | To monitor the trends and determinants of non-communicable diseases and evaluate progress in their prevention and control |
